# The Anæsthetic Action of Chlorine

**Published:** 1881-07

**Authors:** 


### THE ANÆSTHETIC ACTION OF CHLORINE.

Ten years ago Cameron published a singular case of a sailor who died comatose, after having slept in a cabin containing a quantity of chloride of lime. In the post-mortem it was noticed that a strong odor of chlorine was exhaled from the ventricles of the brain when they were opened, although the autopsy was only made thirty hours after death. Binz has recently observed a similar effect in the case of rabbits, and his researches show that chlorine, bromine, and iodine, when inhaled, paralyze the nerve centres, apparently by a direct action on the protoplasm of the nerve cells, and cause death by cessation of the respiration, not of the heart. It used to be taught that chorline when inhaled is quickly transferred into chloride of sodium by combining with the alkali of the blood. It seems, however, that, as Cameron's case teaches, the chlorine may remain for a long time in contact with the lymph and blood without undergoing this transformation.—*Lancet.*

